# Airfoil-shaped filament feed spacer for improved filtration performance in water treatment

**DOI:** 10.1038/s41598-023-37885-5

**Published:** 2023-07-04

**Authors:** Adnan Qamar, Sarah Kerdi, Johannes S. Vrouwenvelder, Noreddine Ghaffour

**Affiliations:** 1 Water Desalination and Reuse Center (WDRC), King Abdullah University of Science and Technology (KAUST), Thuwal, 23955-6900 Saudi Arabia; 2 Environmental Science and Engineering Program, Biological and Environmental Science and Engineering (BESE) Division, King Abdullah University of Science and Technology (KAUST), Thuwal, 23955-6900 Saudi Arabia

**Keywords:** Environmental sciences, Materials science

## Abstract

Optimal spacer design enhances the filtration performance in spiral-wound modules by controlling the local hydrodynamics inside the filtration channel. A novel airfoil feed spacer design fabricated using 3D-printing technology is proposed in this study. The design is a ladder-shaped configuration with primary airfoil-shaped filaments facing the incoming feed flow. The airfoil filaments are reinforced by cylindrical pillars supporting the membrane surface. Laterally, all the airfoil filaments are connected by thin cylindrical filaments. The performances of the novel airfoil spacers are evaluated at Angle of Attack (AOA) of 10^°^ (A-10 spacer) and 30^°^ (A-30 spacer) and compared with commercial (COM) spacer. At fixed operating conditions, simulations indicate steady-state hydrodynamics inside the channel for A-10 spacer, while an unsteady state is found for A-30 spacer. Numerical wall shear stress for airfoil spacers is uniformly distributed and has a higher magnitude than the COM spacer. A-30 spacer design is the most efficient in ultrafiltration process with enhanced permeate flux (228%) and reduced specific energy consumption (23%) and biofouling development (74%) as characterized by Optical Coherence Tomography. Results systematically demonstrate the influential role of airfoil-shaped filaments for feed spacer design. Modifying AOA allows localized hydrodynamics to be effectively controlled according to the filtration type and operating conditions.

## Introduction

Over the past decade, the freshwater shortage has been continuously rising and putting tremendous pressure on existing freshwater resources^1^. In addition, the recent spreading of coronavirus pandemic, with the ability to infect water for days to weeks^2^, places tremendous stress on producing safe drinking water. Membrane filtration technologies such as reverse osmosis (RO), nanofiltration (NF), and ultrafiltration (UF) have gained attention due to their potential to yield a high quantity and safe drinking water with reasonable operating costs^3^. However, the accumulation of (bio)fouling on the membrane surface ruins filtration performance and deteriorates water quality^4^. Therefore, controlling the (bio)fouling growth is essential for greater water productivity while minimizing energy consumption. The prevention of bacterial growth by smartly designing filtration module components constitutes a straightforward and eco-friendly approach. Focusing on designing an optimal feed spacer in spiral-wound modules (SWM) has recently gained significant thrust to enhance water productivity, reduce (bio)fouling growth, and lower energy consumption^5,6^.

The feed spacer in SWM supports mechanically the membrane leaves and promotes fluid unsteadiness associated with local shear rate, which improves the mass transfer and ultimately tackles bacterial growth^7–9^. However, there is a limiting value of the shear rate, above which bacterial attachment over the membrane is favored, which tarnishes the filtration efficiency and increases the pressure drop in the feed channel^5^. As such, the alteration of hydrodynamics due to the feed spacer integration might negatively influence the filtration process if its design is not well-optimized^5^. Thus, identifying an optimal spacer micro-structure remains, so far, challenging for improving the filtration process^10,11^.

In recent years, the development of 3D-printing technology has contributed to innovative feed spacers with high versatility and more complex geometry^8^. 3D-printing technologies or additive manufacturing are advanced processes that rely on creating physical objects from Computer-Aided Design (CAD) models by adding layer-by-layer materials^12^. 3D-printed spacers were developed by modifying the commercial spacer geometric characteristics^13–16^ or by producing novel micro-structured designs^8,17–23^. Among the recently developed spacer designs, triply periodic minimal surface (TPMS) designs^8,18^, uniform sinusoidal configurations^22^, honeycomb-shaped^23^, perforated^20^, column^17^, and helical^19^ spacers exhibited a potential to alleviate the membrane fouling and improve in the water productivity in lab-scale filtration units. However, some limitations including design complexity and mechanical strength weakness impede their implementation in industrial plants.

The present study proposes a novel spacer design based on airfoil cross-sections. Airfoil shapes are commonly utilized for aircraft wings, wind turbines, and turbomachinery applications. The airfoil cross-section has a curved upper and lower surface optimized to produce a favorable ratio of lift (vertical force component) and drag (horizontal force component) forces achieved by adjusting the angle of attack (AOA). The AOA of an airfoil micro-structure is defined as the angle formed by the chord line and the incoming fluid flow. Conventionally, the feed spacer filament design has circular cross-sections, and the most recent design modifications^24^ manipulate micro-structure or dimensions around the circular shape configuration. Any change in the spacer filament alters the porosity of the filtration channel, which significantly modifies the channel pressure drop, local hydrodynamics, and shear stress level on the membrane surface. It results in a propensity to either support or oppose the filtration efficiency in terms of energy utilization or permeate flux production. Thus, in the development of feed spacer design with circular filaments, it is challenging to isolate the effect of porosity from filtration performance parameters.

A novel feed spacer design is proposed having airfoil-shaped filaments with NACA1410 profile^25^. To the best of our knowledge, the only attempt at using airfoil shape in filtration technologies was the attachment of airfoil discrete-object features to the membrane surface with the aim to numerically optimize the feed channel thickness for increased membrane packing capacity^26^. Our airfoil spacer design holds the inherent advantage that it can manipulate filtration performance by altering the localized hydrodynamics by adjusting the AOA of the airfoil without affecting the channel porosity. A ladder-type positioning is proposed to control the AOA effectively, with filament cells supported by cylindrical pillars at four corners. Ladder-type orientation is already known to produce better filtration performance^27^. Therefore, effectively using this configuration is ideal as it will aid in precisely controlling the AOA of the spacer filament to influence the filtration conditions inside the channel.

In the present study, we first designed and manufactured the airfoil spacer for two AOA (10^◦^ and 30°), denoted as A-10 and A-30, respectively, using 3D-printing technology. The proposed airfoil spacer designs are then numerically evaluated at an elemental level and compared to a commercial spacer, denoted as COM. Then, the spacers are experimentally investigated in a cross-flow lab-scale UF setup in terms of permeate flux production and energy consumption. Their potential in reducing the biofouling development on membrane surface was further *in-situ* evaluated using Optical Coherence Tomography (OCT).

## Materials and methods

### Geometric characteristics of airfoil spacers

**Figure 1:**
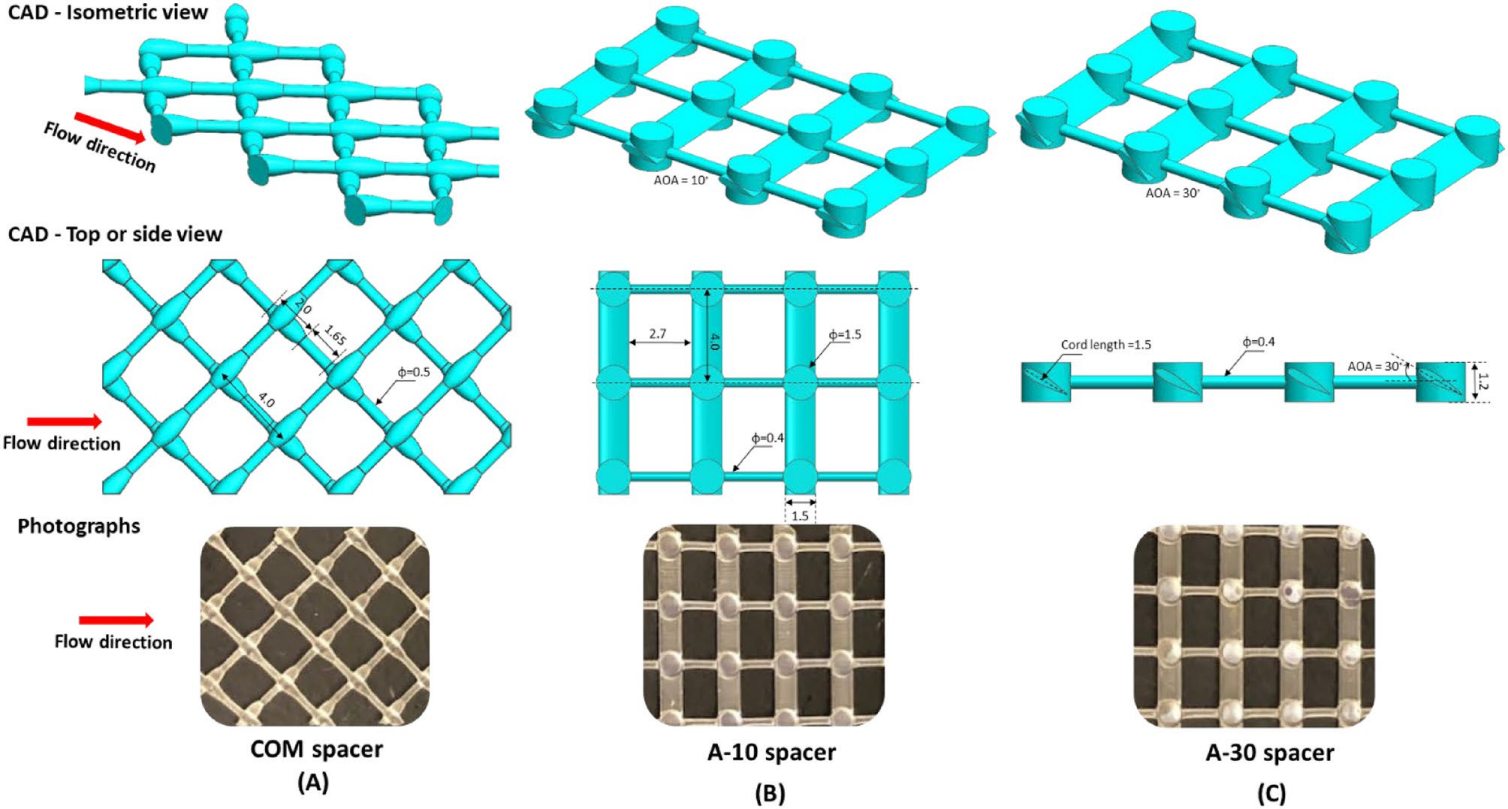
CAD designs and photographs of the 3D-printed spacers: COM spacer (**A**), A-10 spacer (**B**), and A-30 spacer (**C**). The red arrows represent the fluid flow direction. The dimensions are in mm.

Two airfoil spacers (A-10 and A-30) and one commercial spacer (COM) were designed by utilizing CAD models on SolidWorks software (Dassault Systemes SolidWorks Corporation, version 2018). Their dimensions are summarized in Fig. 1. The two designed airfoil spacers had a ladder orientation. Their design consists of two parallel airfoil-type filaments connected by thin cylindrical filaments and reinforced by pillar-type nodes supporting the membrane. The same geometric characteristics were maintained for airfoil spacers except for AOA of the airfoil filaments facing the incoming flow. The inclination angles were 10° and 30° for A-10 and A-30 spacers, respectively. The thickness of all spacers was similar and fixed to fill the channel height of 1.2 mm. The spacers were manufactured layer by layer (layer thickness = 50 µm) via photopolymerization of acrylate monomers (wavelength = 405 nm) by utilizing a 3D-printer based on a Low Force Stereolithography (LFS) technology (Formlabs, model Form 3).

### Synthetic bacterial solution

The prepared feed solution contains 4 g of Bacto Yeast Extract (Extract of Autolysed yeast cells, Becton Dickinson and Company) dissolved in 1 L of Red Sea water and incubated at 30 °C for 24 h to exacerbate the growth of bacteria^5^. After incubation, a volume of 3 L of Red Sea water was added to the enriched bacterial solution. The final feed volume was stirred persistently at 200 RPM with a magnetic stirrer (IKA, KS 4000 i control) at room temperature and employed to feed simultaneously all the flowcells. Regular addition of yeast extract solution (1 g/L) was carried out to maintain a total feed volume of 4 L throughout the UF process.

### Lab-scale UF setup

**Figure 2:**
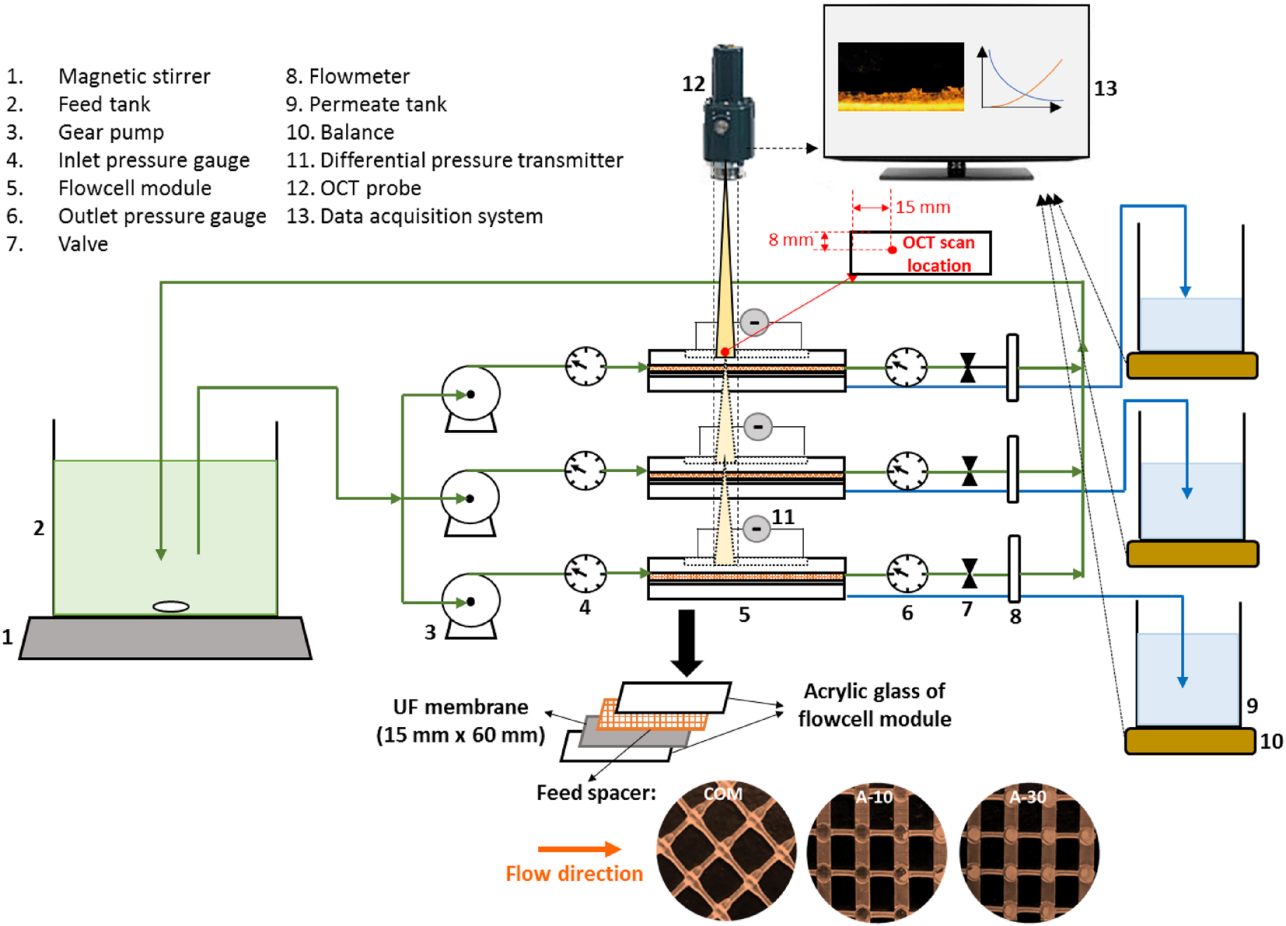
Experimental UF setup associated with the various utilized instruments*.*

The performances of airfoil spacers were experimentally evaluated in UF process conducted for 72 h. The experimental lab-scale apparatus is described in Fig. 2. Applying the same operating conditions and feed solution, UF tests of all spacers (A-10, A-30, and COM) were conducted simultaneously. A flowcell in a cross-flow mode was employed for each tested spacer. Coupons of the spacer and UF flat sheet membrane (Sterlitech polyethersulfone, molecular weight cut-off of 100 kDa) with an active surface area of 900 mm^2^ were integrated into the feed channel having a height of 1.2 mm. This latter was fed by the prepared feed solution using a gear pump (Cole-Parmer, model n° 75211-70, head: N23). The concentrate solution was recirculated to the feed tank. At the inlet and outlet of the flowcell, two pressure gauges (Ashcroft Inc., model n° 1005) were placed in each filtration line to monitor the hydraulic applied pressure (P = 1 bar). A flowmeter (Dwyer, model n° RMB-SSV) was installed at the output to control the desired volumetric flow rate (Q) of 200 mL/min which corresponds to an average inlet feed velocity (U_0_) of 0.185 m/s. The resulting cross-flow velocities (U) (i.e., determined by dividing U_0_ by the filtration channel porosity) in the different spacer-filled channels containing COM and airfoil spacers were 0.208 m/s and 0.231 m/s, respectively. The operating flow rate and hydraulic pressure imposed inside the filtration module were set by using a valve (Swagelok, model n° SS-1VS4) located at the feed outlet. The weight of collected permeate flux was recorded automatically every 5 min through a digital balance (Mettler-Toledo, model n° MS3002S) connected to a data acquisition system (National Instruments, LabVIEW). The initial pressure drop arising from the spacer design was measured across the filtration channel by using a differential pressure transmitter (Omega Engineering, model n° PX5200 M5091/0112) before the onset of the filtration process.

### Biofouling characterization by OCT

Optical Coherence Tomography (OCT) (Thorlabs, Hyperion) was applied to visualize and quantify the biofilm developed in the filtration channel. Three-dimensional (3D) OCT images were taken on membrane surfaces for young and mature biofilms at 24 h and 72 h of filtration progress, respectively. The OCT probe was fixed at the exact locations (8 mm × 15 mm from the feed inlet) of the fouled membranes equipped by the tested spacers. For all images, the same scanned area of 0.49 mm × 0.44 mm was kept with a depth of 1.35 mm along the x–z plane direction and a resolution of 496 × 145 × 167 pixels. Similarly, scan parameters were unchanged: A-Scan rate = 127 Hz, central wavelength = 930 nm, and refractive index = 1.35.

The acquired 3D-OCT images were post-processing using *AVIZO* software (Field Electron and Ion Company, Hillsboro, OR, USA), where multi-sequences (i.e. volume rendering, colormap intensity, and color system adjustment) were implemented to achieve a clear biomass/membrane structural visualization. The biomass volumes developed on the membrane surface were determined by processing 3D-OCT images by multi-sequence steps via *AVIZO* including interactive thresholding, volume rendering, membrane structure subtraction, image segmentation, and statistic calculation of biomass volume by selecting the pixel zones corresponding to the biomass^28^.

### Calculation of filtration parameters

The performance of airfoil spacers in the lab-scale UF system was evaluated by the flux production and energy consumption relative to the commercial spacer. The related filtration parameters were calculated following Eqs. (1), (2), (3), (4), (5), (6) and (7).

The produced permeate flux J (LMH) was calculated by;

1 $$J = \frac{\Delta m}{{A.\Delta t}},$$

where m (kg) is the weight of the produced water, A is the active surface area of the membrane (m^2^), and t (h) is the filtration time.

The total resistance R_t_ (m^−1^) represented the sum of resistances arising from the membrane structure and the fouling layer developed on the membrane surface. It was determined by the following equation;

2 $$R_{t} = \frac{P}{\mu .J}.$$

For this equation, P (mPa) is the applied transmembrane pressure and µ (mPa.s) represents the fluid feed viscosity.

The percentages of flux and total resistance improvement of airfoil spacers were assessed relative to the commercial spacer and calculated by;

3 $$\% J = \frac{{J_{Airfoil} - J_{COM} }}{{J_{COM} }} \times 100,$$

4 $$\% R_{t} = \frac{{R_{t(COM)} - R_{t(Airfoil)} }}{{R_{t(COM)} }} \times 100,$$

where J and R_t_ values correspond respectively to the flux and total resistance recorded at steady-state conditions.

The porosity (ф) of the filtration channel equipped by each tested spacer was determined as follows;

5 $$\phi = 1 - \frac{{V_{spacer} }}{{V_{channel} }},$$

where V_spacer_ (m^3^) and V_channel_ (m^3^) are the spacer volume and the filtration channel volume, respectively.

The initial pressure drop gradients resulting from the spacer integration in the feed channel were defined by ΔP/ΔL (mbar/m), where ΔP (mbar) is the differential pressure over the feed channel measured by the differential pressure transmitter (as depicted in Fig. 2) and ΔL (m) is the feed channel length.

The energy consumption E (kW) was defined as the energy consumed by the overall filtration system to produce the permeate flux. It was determined by the feed and permeate volumetric flow rates associated with the pressure drop related to the spacer design^17^. The energy consumed by the permeate side was assumed negligible as the circulation of permeate flux from the flowcell to the permeate tank was essentially governed by the gravitational force. E (Watt) was estimated as follows;

6 $$E = \frac{Q \times \Delta P}{\eta },$$

where Q (m^3^/s) is the volumetric feed flow rate, ΔP (N/m^2^) is the initial feed channel pressure drop, and ɳ represents the feed pump efficiency which is assumed full (ɳ = 100%) for all UF experiments.

The specific energy consumption SEC (kW.h/m^3^) was considered as the energy expended by the filtration system to yield a unit flux amount^17^ given by;

7 $$SEC = \frac{E}{{Q_{P} }}$$

where Q_p_ (m^3^/h) represents the volumetric permeate flow rate at steady-state conditions.

### Numerical approach

The filtration channels' hydrodynamics with different tested spacers were calculated using Direct Numerical Simulations (DNS) with flow settled to laminar state (steady or unsteady) to resolve the spatial and transient fluid flow distribution. The computations were performed for two whole and two halves spacer cells. The used numerical approach was able to solve Navier–Stokes equations accurately without integrating any turbulent models. The utilized numerical methodology is presented in our previous study^29^. The particulars of the computational domain and mesh convergence can be found in the Supplementary Information (SI) document. The fluid was assumed to have Newtonian properties and the membrane surface was considered a solid wall. No-slip boundary condition was applied by assuming impermeable walls, as velocity components near the membrane for a small computational domain are negligible^29^. Considering the symmetry and replication of spacer cells, periodic boundary conditions were imposed in the spanwise direction.

All the governing equations and appropriate boundary conditions were solved in a finite-volume framework via the commercial solver ANSYS Fluent 2020 R1 (ANSYS, Inc., Canonsburg, PA, USA). The computational domain was discretized into 12.6 million control volumes. Second-order formulations were utilized for spatial and temporal discretization. The convective term in the pressure formulation was discretized by applying QUICK (Quadratic Upstream Interpolation for Convective Kinematics)^30^ approach. The PISO (Pressure Implicit with Split Operator)^31^ algorithm was used to couple the pressure–velocity. Due to a large number of the discretized equations, all the simulations were performed by using 1024 cores on Intel Haswell Processor (2 CPU socket per node, 16 cores per CPU, 2.3 GHz with 128 GB of memory per node) as offered by the KAUST Supercomputing facility (SHAHEEN II)^32^.

## Results and discussion

Essential hydrodynamics simulations followed by experimental UF performance evaluation were meticulously investigated for airfoil spacers and compared to the commercial spacer.

### Numerical performance of airfoil spacers

Numerical analysis was achieved to provide an indicator of filtration performance before conducting the filtration experiments.

### Pressure drop determination of airfoil spacer-filled channel

Cross-flow pressure drop in a spacer-filled channel is a vital parameter while designing a feed spacer, as specific energy consumption of the filtration system is greatly dependent on geometric spacer characteristics^17,33^. A more significant initial pressure drop would require higher energy consumption from the pump to push the feed solution through the membrane module. This scenario becomes more impactful when biofouling occurs inside the channel with the filtration evolvement and increases the pressure drop resulting in higher energy consumption. Thus, an optimal spacer design offers a low-pressure drop of the filtration channel. Simultaneously, the appropriate spacer design generates unsteady local hydrodynamics, which is not favorable for biofouling growth^5^.

**Figure 3:**
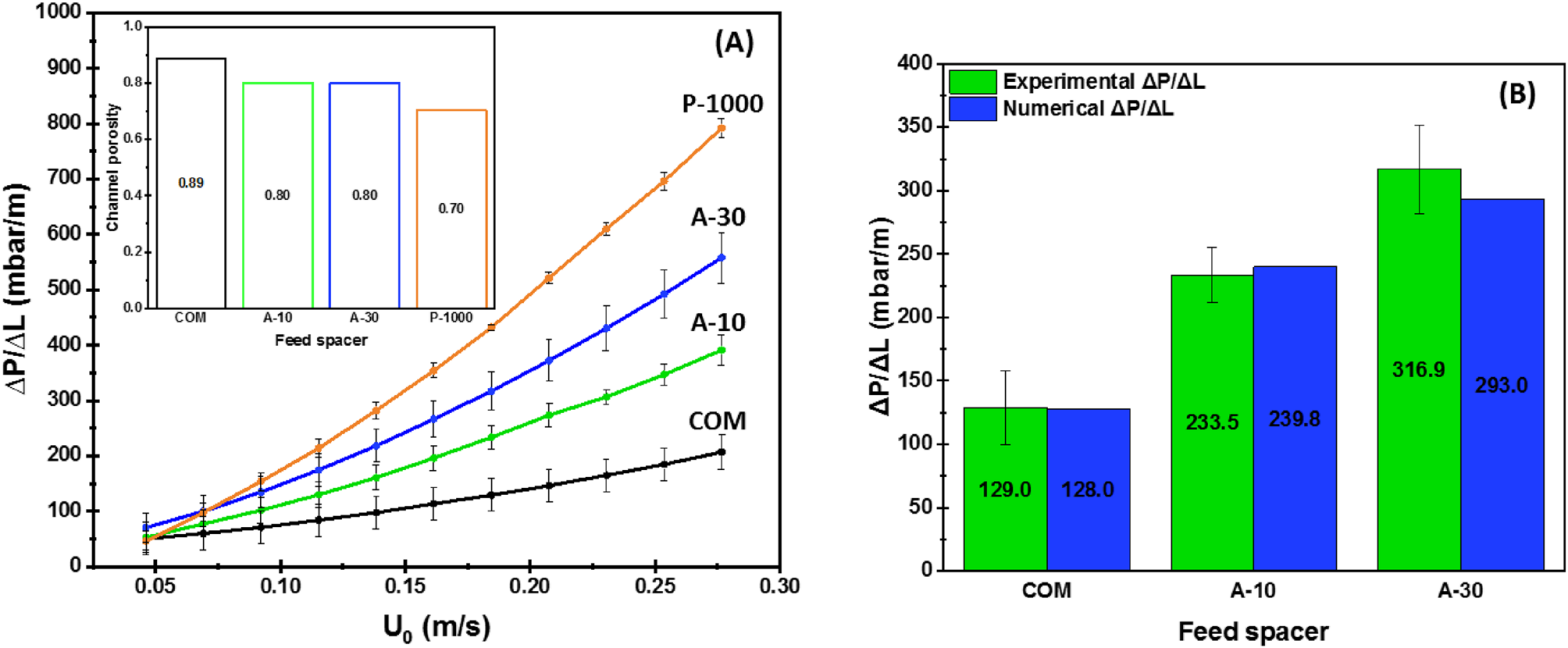
Experimental variation of the differential initial pressure drop gradients as a function of inlet feed velocity along with the porosity of channels integrated with the different spacers before the biofouling growth (**A**), comparison of their corresponding experimental and numerical differential pressure drop gradients at average inlet feed velocity of U_0_ = 0.185 m/s (**B**).

Before the onset of UF process, cross-flow channel pressure drop was estimated for each spacer to investigate the impact of spacer design on building the pressure loss across the filtration channel without the interference of fouling effect. Figure 3A shows the comparison of pressure drop gradients as a function of channel average inlet feed velocity for the airfoil spacers. COM spacer and pillar spacer developed by Ali et al.^17^, having the same channel thickness of 1.2 mm, are presented for comparison. The COM spacer had the minimum pressure drop gradient at the measured range of inlet feed velocity (U_0_ = 0.046–0.277 m/s, Re = 55.2–332.4) followed by airfoil spacers and then pillar spacer. A-10 spacer had a lower pressure drop than A-30 spacer. Although there was no difference between the airfoil spacer dimensions, A-30 spacer produced more hydrodynamic drag force than the streamlined A-10 spacer resulting in higher pressure drop gradient. As the incoming feed flow enters the filtration channel facing the airfoil filament with higher AOA, A-30 spacer tends to obstruct the incoming flow more prominently than A-10 spacer causing a greater fluid drag force.

At fixed channel height, the spacer design which produces a lower pressure drop has a high channel porosity and thereby lower cross-flow velocity for a constant inlet feed velocity^34^. In agreement with this approach, Fig. 3A showed that as the channel porosity increased, the pressure drop gradient decreased for the same channel height. Although the commercial spacer design revealed lower pressure drop than the airfoil design, a lower cross-flow velocity was further promoted in the commercial spacer-filled channel (U = 0.208 m/s against 0.231 m/s for the airfoil design). Consequently, a higher permeate flux production was anticipated using airfoil spacers regardless of AOA value. Moreover, airfoil spacers having the same channel porosity (i.e. the same cross-flow channel velocity) significantly altered the pressure drop because of the different hydrodynamic drag forces generated by the variation of AOA (10° and 30°). Interestingly, airfoil spacer design can manipulate the channel pressure drop gradient and thereby control the energy consumption by only varying the local hydrodynamic drag, which is induced by modifying AOA without any disturbance of the cross-flow velocity in the filtration channel.

Numerical calculations were carried out on commercial and airfoil spacers for validation of the numerical model. Figure 3B compared the numerical and experimental pressure drop gradients at U_0_ = 0.185 m/s (average inlet feed velocity applied in UF tests). The numerical model accurately captured the experimental pressure drop for COM spacer and reasonably well for A-10 with an error deviation of less than 3%. However, the comparison varied by 7% for A-30. This larger variation between experimental and numerical pressure drop values for A-30 spacer was primarily associated with unsteady hydrodynamic conditions contrarily to the other spacers (subsequently demonstrated in “Hydrodynamic conditions at airfoil spacer elemental level” section). In unsteady hydrodynamic conditions, spatial and temporal flow averaging are critical and often result in comparison with relatively more significant errors^35,36^ as seen for A-30 spacer. In addition, due to minor dimensional discrepancies while 3D-printing, the numerical meshing of intricate spacer designs along with the reading error of equipment involved in the experiments might contribute overall to validation errors.

Although spacer design primarily focuses on increasing the porosity of the channel by reducing the filament cross-section, the chance of tripping localized hydrodynamics to unsteady conditions is also diminished, which favors a higher potential of biofilm growth^5,37^. As observed, COM spacer produced a lower initial pressure drop than airfoil spacers. However, COM spacer design is known to have steady hydrodynamic conditions^38^ at typical filtration operating conditions, and thus higher biofouling is favored^39^. With airfoil spacer design, the hydrodynamic drag or pressure drop can be well controlled by varying the AOA without altering the channel porosity, which offers an additional control parameter while designing novel and efficient feed spacers.

### Hydrodynamic conditions at airfoil spacer elemental level

Understanding the local hydrodynamics at an elemental level is an essential part of designing the new spacer. If the fluid inlet velocity is kept constant, the spacer design significantly alters the local velocity inside the channel, which produces different hydrodynamic shear stress on the membrane surface^5,17,33^. High spatial shear stress on membrane aids in minimizing the concentration polarization^40^. However, it is detrimental for pre-filtration processes (like UF or NF) based on biologically active feeds, where localized high shear stress favors the biofilm formation on membrane surfaces^5^. Thus, depending on the application, an appropriate spacer design must be gauged at an elemental level to significantly boost the filtration performance.

#### Temporal behavior

**Figure 4:**
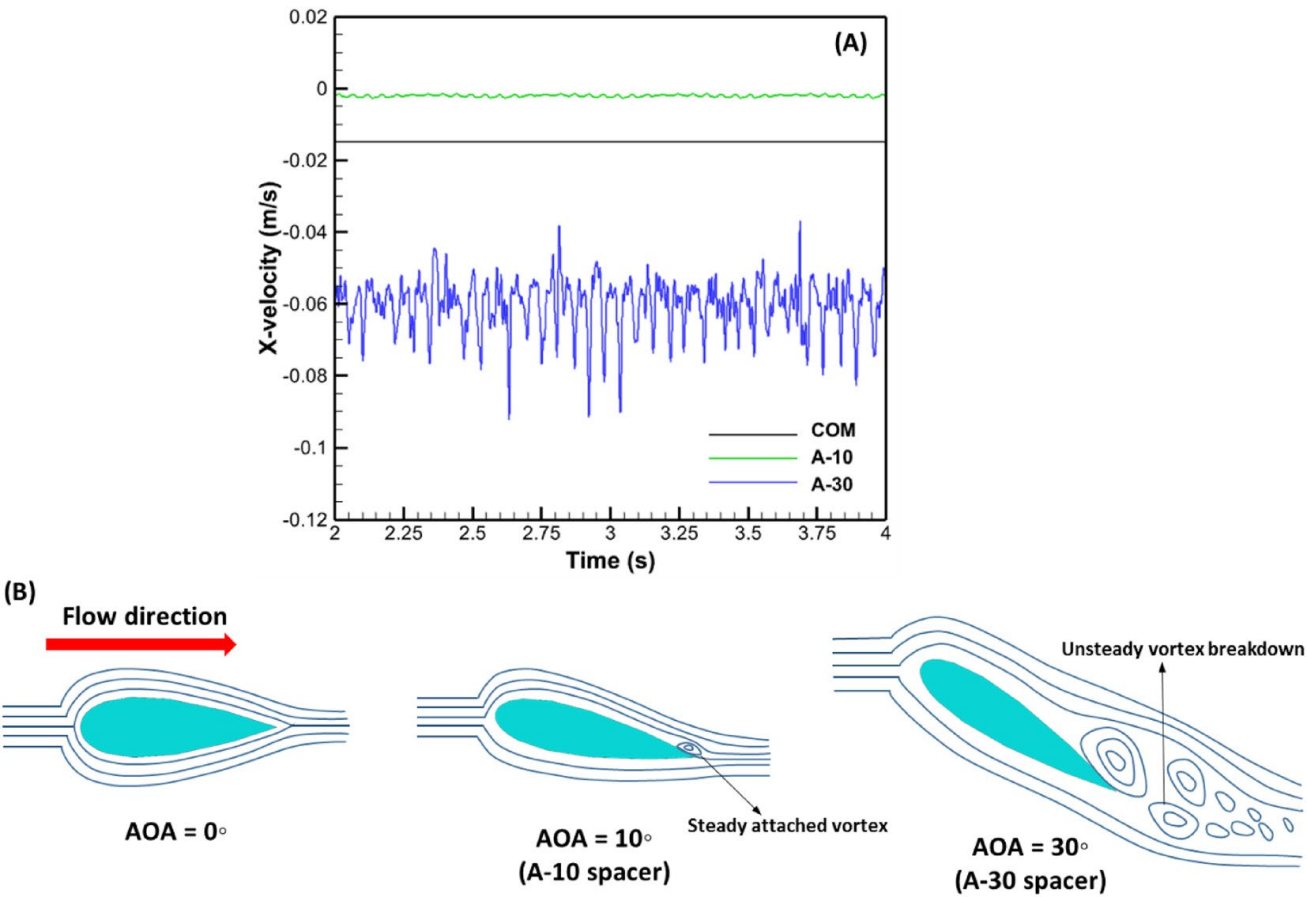
Temporal x-velocity component behavior for numerical probe placed in the computation domain at (X = 8 mm, Y = 0.05 mm, Z = 4 mm) and (X = 10.35 mm, Y = 0.05 mm, Z = 2.56 mm) for airfoil and commercial spacers, respectively (A) and schematic representation of flow streamline for various AOA at low Reynolds number (**B**).

Figure 4A showed the temporal variation of x-velocity as a function of time at a specific spatial location behind the filament intersection inside the computational domain for all spacers. When the flow was fully evolved, it could be seen from the temporal signal of the numerical probe that the flow inside the channel having COM spacer was steady. While for A-10 spacer, the flow was still stable with minimal perturbation in the time signal. On the other hand, A-30 spacer clearly showed a strong unsteady flow inside the channel.

The aerodynamic performance of airfoils and streamlined bodies is a strong function of the laminar-unsteady transition, and consequently, the critical Reynolds number (Re). However, in filtration processes, channel flow velocities (or Re) are mostly fixed due to operational constraints. Thus, to achieve an unsteady flow transition in the filtration channel, AOA is a vital parameter to consider. In fact, aerodynamic studies^41^ at low Re number indicate that the onset to unsteady transition occurs due to the flow separation bubble instigated by shear layer impingement on the upper camber of the airfoil. For AOA < 5^◦^_,_ the flow separation bubble is not visible, and the incoming flow detaches at the leading edge by creating a stagnation point (highest pressure point). Under this scenario, the reattachment of the streamlines occurs close to the sharp trailing edge of the airfoil (Fig. 4B). As the AOA increases, the detachment point moves downward toward the lower camber, while the flow reattachment point moves upward towards the airfoil's upper camber. Depending on Re number, the flow separation bubble appears for AOA > 5°. At a very low Re number, the formed separation bubble near the trailing edge provides small perturbations in flow. If the AOA is further increased, the separation bubble destabilizes and ultimately leads to Von-Karman type shedding^42^. At a reasonable Re number, the flow fully transits to a unsteady state with intensity depending on Re number.

#### Localized spatial flow field

**Figure 5:**
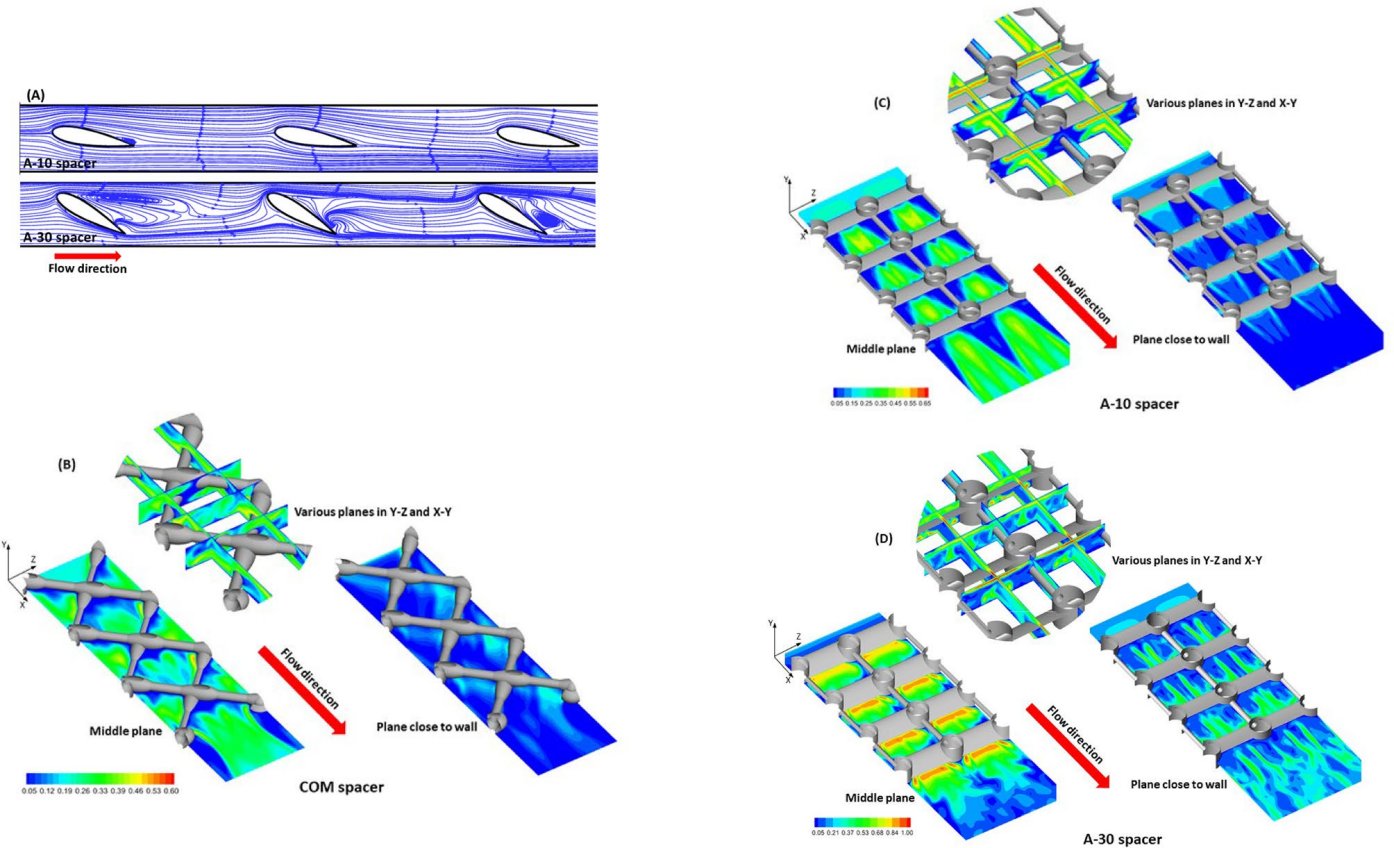
Streamtrace patterns extracted using numerical calculations on X–Z plane for A-10 (top) and A-30 (bottom) spacers depicting steady and unsteady flow development inside the filtration channel (**A**), spatial velocity magnitude contours at various locations for COM spacer (**B**), A-10 spacer (**C**), and A-30 spacer (**D**). The left image of each group is the contour plot on X–Y plane in the middle of the channel and the right image is the contour at the bottom plane very close to the wall. The middle image is taken for contour at various Y–Z and X–Z planes.

As aforementioned, similar hydrodynamic transitions for the airfoil spacer filaments were observed. Figure 5A showed the streamlines at the central plane of the airfoil spacers. Clearly, a small separation bubble was visible at the trailing edge of A-10 spacer, while for A-30 the separation bubble grew and moved away from the trailing edge and ultimately busted into unsteady sheading, as seen in the time signal of the numerical probe (Fig. 4A).

To investigate the local hydrodynamic conditions occurring inside an elemental cell, flow velocity magnitude contours were plotted at various slices inside the computational domain as presented in Figs. 5B–D for different spacers. For all spacers, the velocity magnitudes picked up locally under the spacer filaments satisfying the mass and momentum conservation, which were proportional to the filament's clearance regions and cross-section shapes^5,17^.

The commercial spacer produced an asymmetric flow field (Fig. 5B), owing to a non-woven design. In the mid-plane, a low-velocity magnitude was observed behind the filaments due to recirculating vortices, which were typically seen behind the bluff body flows^43^. At the center of the spacer filament cell, the incoming flow segregated by the asymmetric filament joint merged and picked up velocity. At the plane close to the wall, a low-velocity magnitude was observed.

For A-10 spacer (Fig. 5C), the incoming flow was detached by the leading edge of the airfoil cross-section, and due to limited space between the airfoil camber to the top and bottom walls, local flow velocity increased and reached a higher velocity magnitude (~ 0.6 m/s) to satisfying the mass conservation. For A-30 spacer, the gap was further reduced. Thus, a significant increase in the flow velocity (~ 1 m/s) was noted for this case, especially near the wall (Fig. 5D). At the mid-channel plane for A-10 spacer (Y–Z and X–Y planes), a higher velocity magnitude was observed under steady conditions. In comparison, the velocity magnitude was lower for A-30 spacer but unsteady effects increased along the flow direction from one cell to another. In principle, it was expected that if more flow cells were utilized, the channel would ultimately transit to an unsteady state at an AOA of 30°. The least flow magnitude was detected behind the pillar attached by a cylindrical filament for both cases. However, A-30 spacer had an additional advantage gained through unsteady behavior. The mixing and shedding of vortices (including the vortices generated by cylindrical pillars) provided enough momentum to actively sweep the low-velocity zones. This can easily manifest in assuming that foulant deposition has a lower chance to settle for A-30 than A-10 spacer.

In conclusion, higher cross-flow velocity is provided by airfoil spacer due to the lower channel porosity created by the design, which predicts an increase of permeate flux relative to the commercial design. Moreover, a raise of AOA in airfoil design (i.e. increase of pressure drop) is needed to generate a flow unsteady state in filtration channel as demonstrated in case of A-30 spacer. Consequently, an improvement of filtration performance and fouling mitigation at the expense of energy consumption are foreseen because of the unsteadiness promoted by AOA raise. Therefore, the determination of specific energy consumption (subsequently discussed in section “Permeate flux production and relative filtration energy requirements”) is crucial to assess the total benefits of using airfoil spacer compared to the commercial spacer.

#### Elemental flow profiles

**Figure 6:**
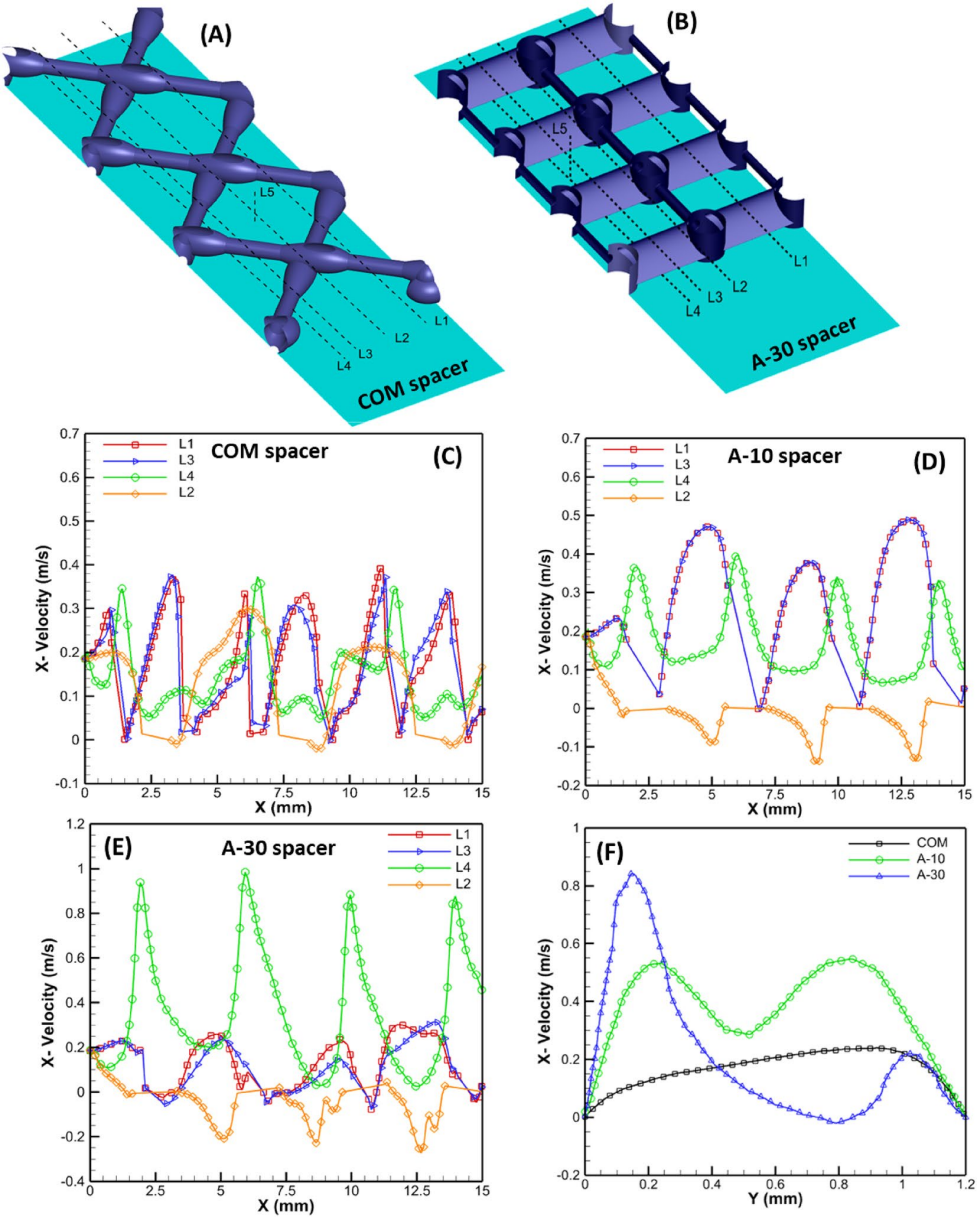
Local elementary flow profiles computed at various locations inside the computation domain. Spatial locations of flow profile at various locations for COM spacer (**A**) and airfoil spacers (**B**), x-velocity component of flow along the flow profile for COM spacer (**C**), A-10 spacer (**D**), and A-30 spacer (**E**), and boundary layer profiles for all spacers at the center of the spacer cell (**F**).

Localized spatial flow profiles were extracted from the 3D computation field at critical locations, as presented in Table 1. For all spacers, spatial lines L1 and L3 were placed at similar locations in two adjacent filament cells in Z-direction at mid-channel height, whereas L2 and L4 were at locations close to the wall (or membrane surface) (Fig. 6A,B).

**Table 1:** Coordinates of the numerical extracted profile lines to access localized flow inside the channel.

Profile lines	Airfoil spacers			Commercial spacer		
	X (mm)	Y (mm)	Z (mm)	X (mm)	Y (mm)	Z (mm)
L1	0–20	0.6	6	0–20	0.6	3.66
L2	0–20	0.05	4	0–20	0.05	2.56
L3	0–20	0.6	2	0–20	0.6	1.21
L4	0–20	0.05	2	0–20	0.05	1.21
L5	8.5	0–1.2	2	10.35	0–1.2	2.56

For commercial spacer, the local hydrodynamics is slightly different than airfoil spacers (Fig. 6C). For lines L1 and L3, the maximum x-velocity magnitude along the streamwise direction reached between 0.3 and 0.4 m/s, slightly less than A-10 spacer (0.4–0.5 m/s) (Fig. 6D). Further, the profiles of L1 and L3 were not coincident (like seen in A-10 spacer), even when the flow inside the channel was steady. This is attributed to the asymmetry produced by the non-woven design. The x-velocity magnitude of the L4 location close to the membrane surface was roughly similar to A-10 spacer. However, the flow profile L2 along the filament intersection had a lower x-velocity magnitude with slight negative x-velocity.

For A-10 spacer (Fig. 6D), the x-velocity profile along the spatial line L1 and L3 overlapped, clearly suggesting the presence of a steady-state in the adjacent flow cells. At spatial location L4, the fluid momentum was diverted towards the wall due to the small AOA of 10^◦^, increasing x-velocity magnitude. Location L4 had a lower velocity magnitude (0.3–0.4 m/s) than L3 or L1 mid location which appeared to have the highest localized velocity ~ 0.4–0.5 m/s in the spacer cells. The lowest velocity was seen behind the pillars at the L2 location, where negative values of x-velocity suggested the presence of a steady vortex that appears to be the potential location for foulant accumulation.

For A-30 spacer (Fig. 6E), the incoming fluid momentum was diverted effectively toward the membrane surface. The spatial location L1 and L3 did not overlap, clearly suggesting unsteady flow presence. A significant increase in flow velocity was achieved at location L4, as the gap between the trailing edge of the airfoil came closer to the wall, reducing the cross-section area of the flow, thus significantly enhancing the local x-velocity magnitude. For this case, the highest velocity was observed for location L4, with values reaching between 0.8–1 m/s. In addition, the flow behind the pillar (L2 location) showed a larger negative x-velocity, with profiles changing in each subsequent spacer cell. It indicates that the vortex behind the pillar was shedding, sweeping the wall behind the pillars. In principle, this is ideal for filtration purposes, as it will not allow foulant formation behind the pillar, leading to enhanced filtration performance.

Contrary to commercial spacer design, at the same location L2 the x-velocity magnitude generally remains negative along the spanwise direction, suggesting the vortex behind the airfoil spacer pillar spans in a larger volume than the commercial spacer. From a hydrodynamics perspective, unsteady vortices are advantageous as they produce oscillatory shear stress on the membrane surface, which is effective for cleaning or delaying biofilm growth^5^.

The boundary layer profiles for the three spacers were also extracted to evaluate the variation of the local velocity along the channel height (Fig. 6F). The boundary layer profile was extracted in the middle of the spacer filament cell along line L5, as depicted in Fig. 6A,B. Local x-velocity magnitudes were found highest for A-30 spacer followed by A-10 spacer and then COM spacer. Increasing the AOA in the airfoil spacer design produces a higher localized velocity field, which can be beneficial for fouling removal and concentration polarization mitigation. However, higher AOA is associated with more hydrodynamic drag, potentially consuming more filtration energy. An optimal AOA would ideally depend on the feed type, filtration process, and process conditions.

#### Spatial shear stress distribution

**Figure 7:**
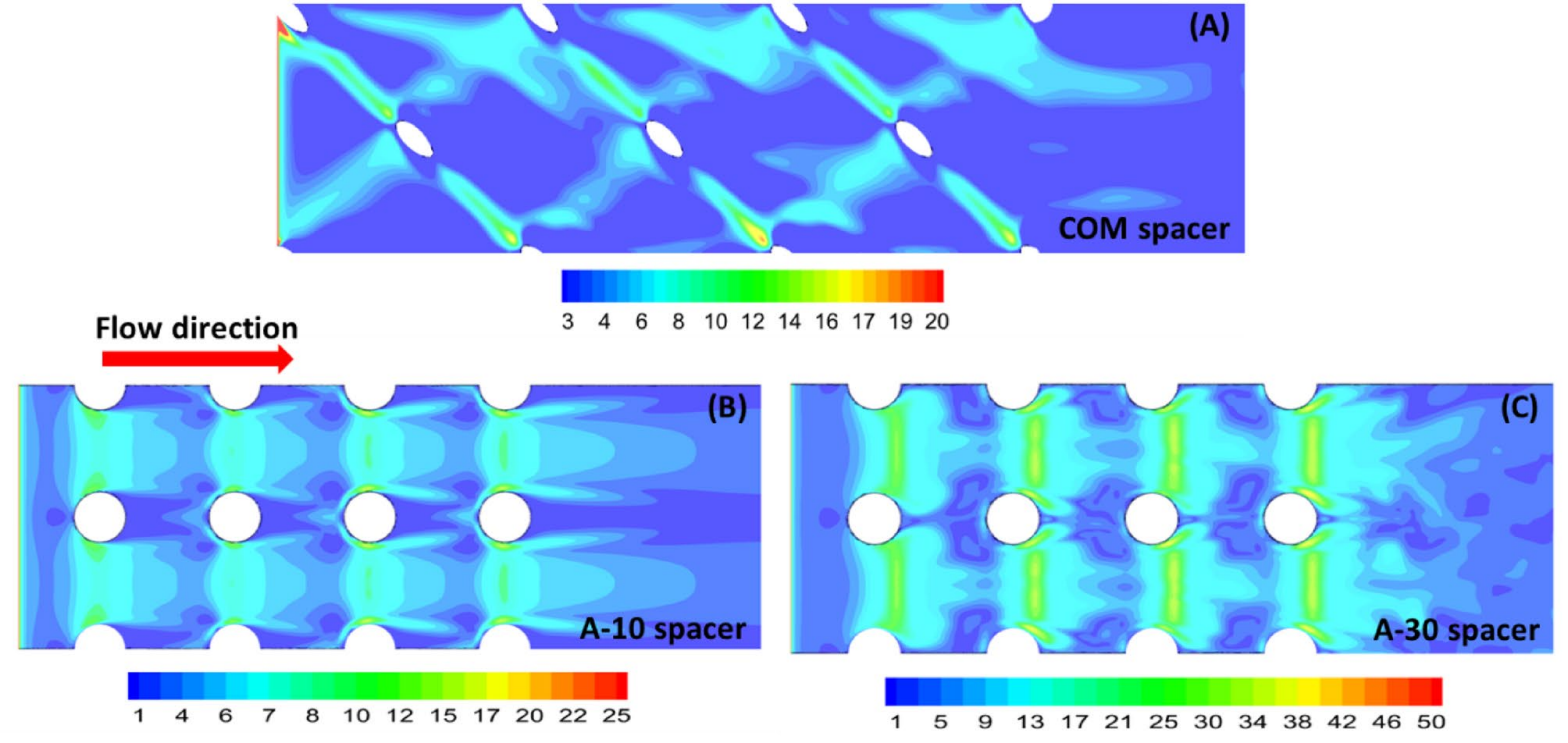
Computational shear stress contours for COM spacer (**A**), A-10 spacer (**B**), and A-30 spacer (**C**) on the bottom wall of the computational domain.

The proposed airfoil spacer is primarily designed to improve filtration performance and inherently control biofilm growth. Thus, understanding the spatial shear stress distribution is essential in a numerical framework. Figure 7 shows the spatial shear stress distribution for all spacers at a uniform inlet velocity of U_o_ = 0.185 m/s.

For airfoil spacers, the shear stress distribution was symmetrically distributed over each spacer cell compared to the commercial spacer. As COM spacer is non-woven, only one layer of filament strand touches the wall (or membrane), while the other filament layer supports the opposite wall leading to asymmetric shear stress distribution on the membrane surface (Fig. 7A). The highest shear stress was seen under the filament for all spacers. For airfoil spacers (Fig. 7B,C), the shear stress was more uniform and evenly distributed. For A-10 spacer, a mild increase in shear stress was visible on the sides of the central pillar (Fig. 7B), along with the spatial location where the trailing edge was present. The magnitude of shear stress under the airfoil filament of A-10 spacer was similar to COM spacer (8–16 N/m^2^). However, the central region of the spacer cell had relatively higher shear stress for A-10 spacer (6–7 N/m^2^), compared to COM spacer (3–4 N/m^2^).

On the other hand, A-30 spacer showed a relatively high shear stress value under the filaments (34–40 N/m^2^). The central region of the spacer cell also had a higher oscillatory shear stress value (10–14 N/m^2^). High values of shear stress on the membrane surface can seed initial biofouling faster^5,37,44^. However, as the localized flow was fully unsteady for A-30 spacer, it would prevent/delay the bacterial growth.

## Experimental performance of airfoil spacers in UF process

After evaluating the elemental performance of the airfoil spacers, they were further experimentally assessed to gauge the UF performance and the anti-biofouling propensity.

### Permeate flux production and relative filtration energy requirements

**Figure 8:**
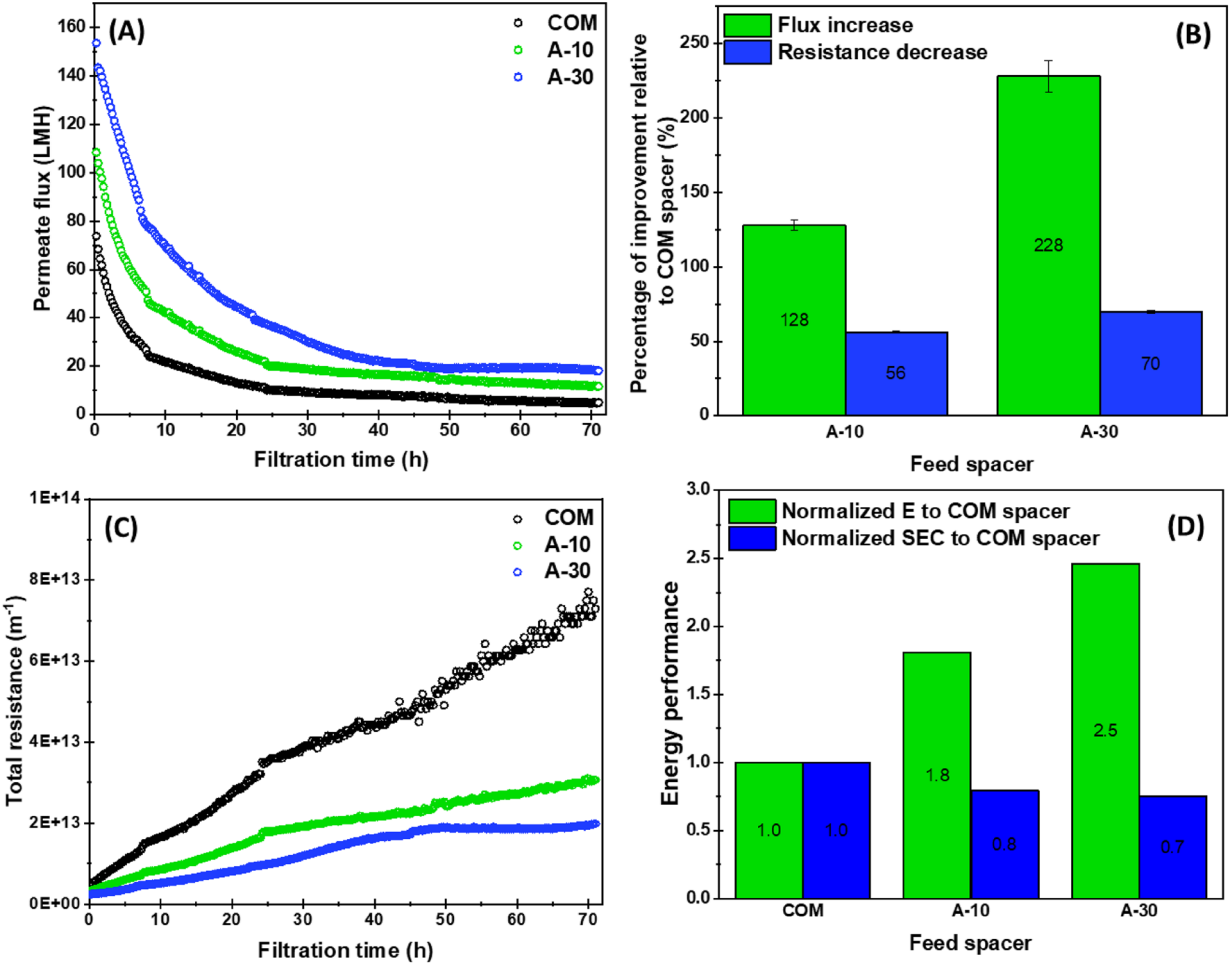
Experimental performances of airfoil and commercial spacers in UF process. Evolution of permeate flux over UF progress (**A**), the percentage improvement of filtration parameters introduced by using airfoil spacers relative to the commercial spacer (**B**), the total resistance evolution as a function of filtration time (**C**), and the energy performance (**D**) for the different tested spacers. For (A), the first flux measurement occurred at 5 min of the filtration process for all curves.

Figure 8 shows the variation of permeate flux, total resistance, and the energy consumed using airfoil and commercial spacers in UF process. For all tested spacers, the flux decline could be described by two essential stages: a sharp decay at the early filtration period followed by a steady-state phase (Fig. 8A). At the early filtration period, the permeate flux decreased rapidly due to membrane pore-clogging associated with deposition of an initial fouling layer on UF membrane surface^45^. With the growth of fouling layer thickness, the flux reduction rate became slower to attain the steady-state approach^46,47^. As observed in Fig. 8A, more filtration time (≈ 45 h) was needed to reach the steady-state flux for A-30 spacer compared to the other spacers. This is most probably attributed to the continuous sweeping away of foulants from the surface^48^. The latter spacer was found to outperform COM and A-10 spacers in terms of steady-state permeate flux. Regardless of the AOA of airfoil spacers, the integration of this novel spacer design within the filtration channel helped to enhance significantly the flux production by 128% and 228% for A-10 and A-30, respectively, relative to the commercial design (Fig. 8B).

The total resistance (R_t_) is defined as the sum of resistances arising from the membrane structure and biofouling cake. As the same UF membrane type was used in all tests, the membrane resistance was assumed constant for all spacer cases. Therefore, R_t_ is considered to reflect the fouling resistance developed over filtration time due to the development of fouling throughout UF tests of the different spacers. Following Eq. (2), R_t_ is inversely proportional to the flux produced at a certain time for fixed operating conditions (pressure and feed solution). Therefore, it has been predicted that the higher the flux produced by a spacer design, the lower the fouling developed on UF membrane. This hypothesis was confirmed by the plot of Fig. 8C where the highest resistance was observed for commercial spacer having the lowest flux, followed by A-10 and then A-30 spacer. The airfoil spacers reduced the biofouling resistance by 56 and 70% relative to the commercial spacer (Fig. 8B). This finding demonstrated that the related biofouling potential was lower for airfoil spacers. Similar to the permeate flux, the resistance trends could be divided into two regions depending on the slope of the resistance curve. For COM spacer, the slope was highest during the first 25 h and gradually shifted to a lower value after this filtration time. Similar trends were observed for A-10 and A-30 spacers, with slope values further reduced.

These findings indicated that the biofouling formation was aggressive in presence of COM and A-10 spacers attributed to the steady nature of hydrodynamics (as discussed in “Hydrodynamic conditions at airfoil spacer elemental level” section) inside the filtration channel^5^. Contrarily, the feed flow unsteadiness generated by A-30 spacer at the elemental level resulted in a substantial delay in biofouling growth on the membrane surface^5,49^.

Applying the same operating conditions, the energy (E) consumed by the filtration system integrated with different spacers is intrinsically correlated to the corresponding initial pressure drop in the feed channel (Eq. (6)). Normalized to the COM spacer consumption, A-30 spacer which had the highest pressure drop gradient (Fig. 3A) was found to consume the highest filtration energy, as seen in Fig. 8D. However, another important factor that should be taken into consideration for the engineering aspect of the spacer design is the hydrodynamic behavior promoted in the filtration channel, which determines the fouling potential and permeate flux production^5,33,50^. Therefore, an optimal spacer design performance is evaluated depending not only on the energy consumed by the filtration system but also on the relative produced permeate flux. The specific energy consumption (SEC) parameter, which correlates the required energy with the produced flux (Eq. (7)) was then examined to provide an accurate and comprehensive evaluation of spacer design performance in the filtration system^17,51^. SEC values were normalized to the COM spacer and found to be lower for airfoil spacers (Fig. 8D). The highest steady-state flux and the lowest fouling resistance (Fig. 8A–C) against the fluid flow equipped by airfoil spacers enhanced the SEC by 23% compared to COM spacer.

### Biofouling characterization by OCT

**Figure 9:**
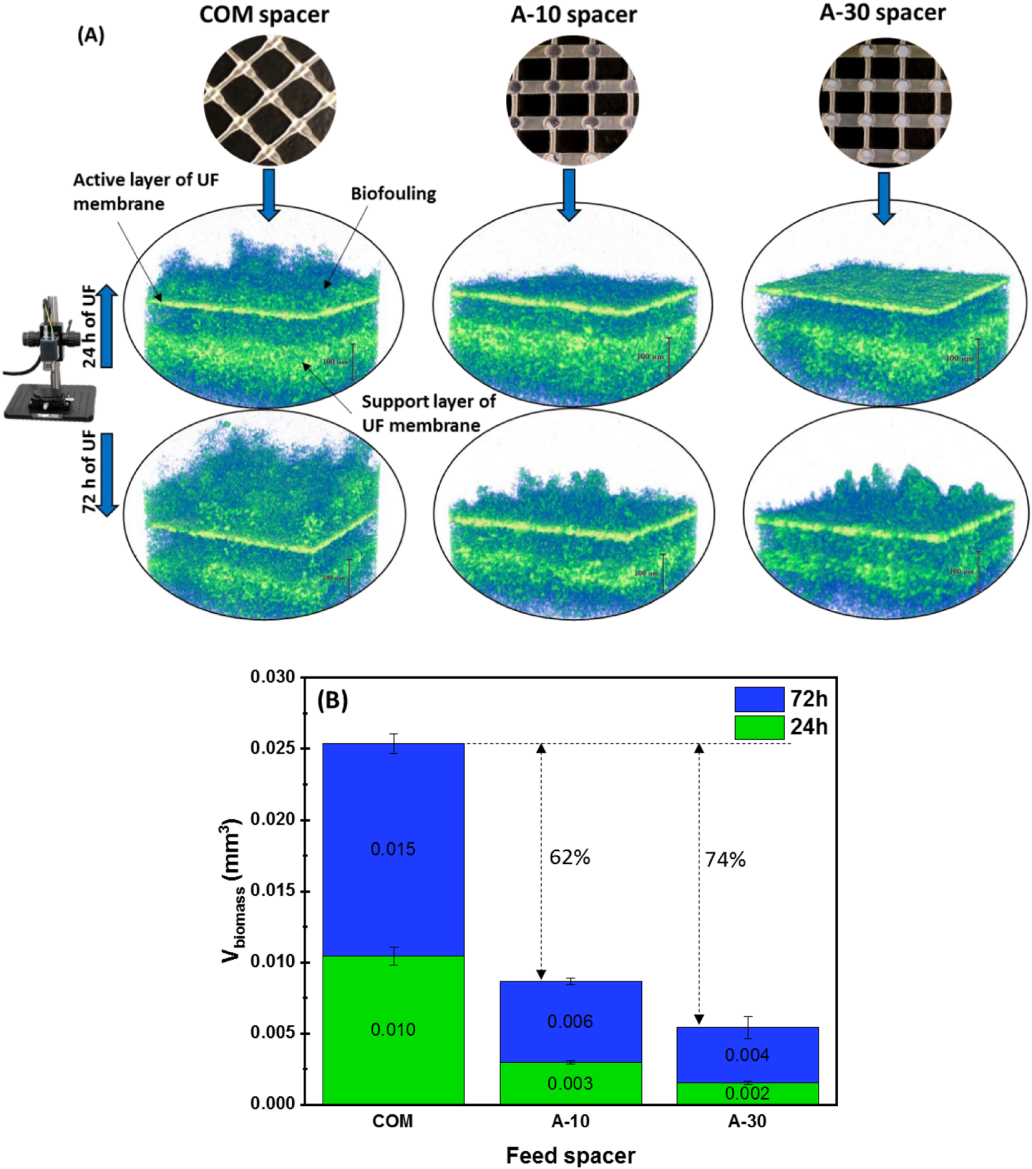
OCT characterization of biofouling developed on membrane surface at 24 h and 72 h of UF test evolvement: biofouling visualization (**A**), and average biomass volume (**B**) in presence of different tested spacers.

Biofouling growth is a detrimental phenomenon in filtration technologies, causing the deterioration of filtration performance and excessive energy cost increase due to the resulting flux decline and channel pressure drop rise^52,53^. Thus, the control of biofouling accumulation represents a significant challenge to consider when engineering a feed spacer with novel characteristics^33^. Therefore, the fouling potential of all spacers was examined by utilizing OCT imaging at early (24 h of UF) and developed (72 h of UF) stages of biofouling formation (Fig. 9).

Irrespective of the filtration time, the *in-situ* 3D-OCT images revealed that the thickest biofouling cake was developed with COM spacer, where a dense and heterogeneous biomass material was visualized covering the entire scanned area of membrane surface (Fig. 9A). The corresponding volumes of biomass at different filtration times were estimated to be 0.010 and 0.015 mm^3^ at 24 h and 72 h, respectively (Fig. 9B). For A-10 spacer, a thin biofouling layer (V biomass = 0.003 mm^3^) was observed at 24 h of UF, whereas the membrane equipped by A-30 spacer was found cleaner (V biomass = 0.002 mm^3^) at the same filtration time. With filtration progress (72 h), a greater biofouling deposition was identified for both airfoil spacers with biomass volumes of 0.006 and 0.004 for A-10 and A-30 spacers, respectively.

The airfoil spacers allowed to mitigate the biofouling development on UF membrane surface by 62% (for A-10) and 74% (for A-30) when compared to COM spacer. The fast bacterial growth in the presence of COM spacer was attributed to the steady nature of hydrodynamics inside the channel as well as the low shear stress in most regions of the membrane (“Hydrodynamic conditions at airfoil spacer elemental level” section). Although steady hydrodynamic conditions were also seen for A-10 spacer, the shear stress produced was relatively higher than COM spacer and uniformly distributed on the membrane surface. This resulted in lower fouling resistance and increased flux production for A-10 spacer compared to COM spacer (Fig. 8A–C) having steady hydrodynamic conditions as well. However, A-30 spacer promoted more fluid unsteadiness in the channel, minimizing the bacterial growth and the fouling resistance, and maximizing the permeate flux production.

## Conclusions

In the present study, novel ladder-shaped symmetric airfoil feed spacers are proposed, designed, and fabricated using 3D-printing technology. The airfoil spacers are systematically evaluated and compared with commercial spacer design (COM) which is prominently used in all spiral wound filtration modules. Two airfoil spacers (A-10 and A-30) are tested mainly by changing the angle of attack (AOA) from 10° to 30°. These spacers are first numerically investigated to elucidate the localized hydrodynamics occurring inside the flow channel. Then, actual 3D-printed spacers were experimentally tested for ultrafiltration process. The outcomes of this study are summarized as follows:

- The higher the AOA, the more considerable hydrodynamic drag is generated, which produces a more significant pressure drop.
- Flow inside the filtration channel for COM and A-10 spacers is found to be steady in nature, while it is unsteady for A-30 spacer producing the highest local velocity.
- Flow separation behind the pillars is observed for airfoil spacers. The vortex for A-30 spacer being unsteady continually produces vortex shedding resulting in better cleaning than COM and A-10 spacer.
- The highest fluctuating shear stress is observed for A-30 spacer resulting in the lowest biofouling growth as confirmed by *in-situ* Optical Coherence Tomography.
- Under the same operating conditions, permeate fluxes are higher for airfoil spacers with a percentage increase of 228% and 128% for A-30 and A-10, respectively relative to COM spacer.
- The specific energy consumption is lower by 23% for airfoil spacers compared to COM spacer.

## Supplementary Information

Supplementary Information.

## Data Availability

The datasets generated during and/or analyzed during the current study are available from the corresponding author on reasonable request.

## Abbreviations

J: Permeate flux
M: Weight
t: Filtration time
A: Membrane active area
P: Transmembrane pressure
R_t_: Total resistance
µ: Fluid viscosity
ф: Channel porosity
V: Volume
ΔP: Channel pressure drop
ΔL: Channel length
Q: Feed volumetric flow rate
Q_P_: Permeate volumetric flow rate
E: Energy consumption
SEC: Specific energy consumption
U_0_: Inlet feed velocity
U: Cross-flow channel velocity
Re: Reynolds number
AOA: Angle of attack
