# Supplementary material for: Airfoil-shaped filament feed spacer for improved filtration performance in water treatment
Source: Sci Rep. 2023 Jul 4;13:10798. doi: 10.1038/s41598-023-37885-5 (PMC10319865; doi:10.1038/s41598-023-37885-5)
Supplement: Supplementary file 1 — Supplementary Information. [file 41598_2023_37885_MOESM1_ESM.docx]

**Supplementary Material**

**Airfoil-Shaped Filament Feed Spacer for Improved Filtration Performance in Water Treatment**

Adnan Qamar^₶a^, Sarah Kerdi^₶a*^, Johannes S. Vrouwenvelder^a,b^, and Noreddine Ghaffour^a,b*^

*^a^ Water Desalination and Reuse Center (WDRC), King Abdullah University of Science and Technology (KAUST), Thuwal 23955-6900, Saudi Arabia*

*^b^Environmental Science and Engineering Program, Biological and Environmental Science and Engineering (BESE) Division, King Abdullah University of Science and Technology (KAUST), Thuwal 23955-6900, Saudi Arabia*

*E-mail: [sarah.kerdi@kaust.edu.sa](mailto:sarah.kerdi@kaust.edu.sa),* [*noreddine.ghaffour@kaust.edu.sa*](mailto:noreddine.ghaffour@kaust.edu.sa)

*^₶^ Equal Contribution*

**Computational approach details**

Direct Numerical Simulations (DNS) at the laminar flow state were used in this work to compute the feed flow interaction with the spacer-filled channel. Although the simulation approach is computationally intensive and challenging for complex geometry, it has the advantage of resolving the smallest (Kolmogorov) temporal and spatial turbulent scales. In addition, no turbulence model is required, which makes computations more reliable and accurate. The numerical computations of direct Navier-Stokes equations ^1^ with incompressible flow assumption were performed.

The computational fluid domains for the airfoil spacer-filled channel were extracted by subtracting the respective CAD designs (same CAD that was used for 3D-printing for A-10 and A-30 spacers) of each spacer with the hollow rectangular channel of 22 mm × 8 mm × 1.2 mm (L × W × H) in SolidWorks software (Version 2020). The computational domain along with numerical boundary conditions utilized in the numerical calculations is depicted in Fig. S1. At the inlet, the flow velocity is specified and set equal to the average channel velocity of U_o_ = 0.185 m/s corresponding to the feed flow rate used in the experiments (Q = 200 mL/min). Periodic boundary conditions are used in the spanwise direction of the channel. The typical flow velocity range for permeate flow is between 1 - 10 μm/s ^2^, which is very low compared to the feed cross-flow (0.05% per spacer unit). Consequently, it is safe to assume an impermeable wall to simplify the computations. This assumption allows us to model top and bottom channel walls with no-slip boundary conditions (V = 0). The outflow is kept at a reasonable distance from the spacer filament, allowing convective disturbances to smoothen out and avoiding any non-physical backflow pressure generation at the exit boundary.

The computational domain was discretized into small control volumes using a polyhedral surface and boundary layer mesh, transforming to hexahedral mesh away from the solid boundary (ANSYS Mosaic) ^3^. Surface polyhedral control volume meshes are faster and more robust to converge within a few iterations resulting in faster and more scalable solutions compared to other different types of meshes ^4^. Before actual simulations, a mesh independence study was accomplished to assess the mesh constraints and to ensure that the discretization errors do not influence the accuracy of the solver. As numerical gradients of physical quantities at the walls are tough to resolve, shear stress and flow velocity at two different spatial locations were examined. The shear stress probe was located at the channel's center on the computational domain's top wall, while the flow velocity probe was located at the channel’s centroid. The simulations were carried out for various meshes as presented in Table S1 by monitoring wall shear and velocity. As the number of mesh points increases, the shear stress and flow velocity converge and the difference in the results is found to be less than 1 % for any mesh greater than 24 million grid points. Therefore, for efficient computation, while maintaining the load balancing, a 24 million mesh was used for all computations to resolve the fluid flow accurately.

**Table S1**. Mesh independence study by monitoring wall shear and flow velocity.

| Total mesh | Minimum mesh size  (μm) | Wall shear  (N/m^2^) | Velocity  (m/s) |
| --- | --- | --- | --- |
| 19 million | 74 | 7.750 | 0.472 |
| 24 million | 55 | 6.100 | 0.419 |
| 30 million | 25 | 6.010 | 0.405 |
| 33 million | 12 | 6.002 | 0.400 |


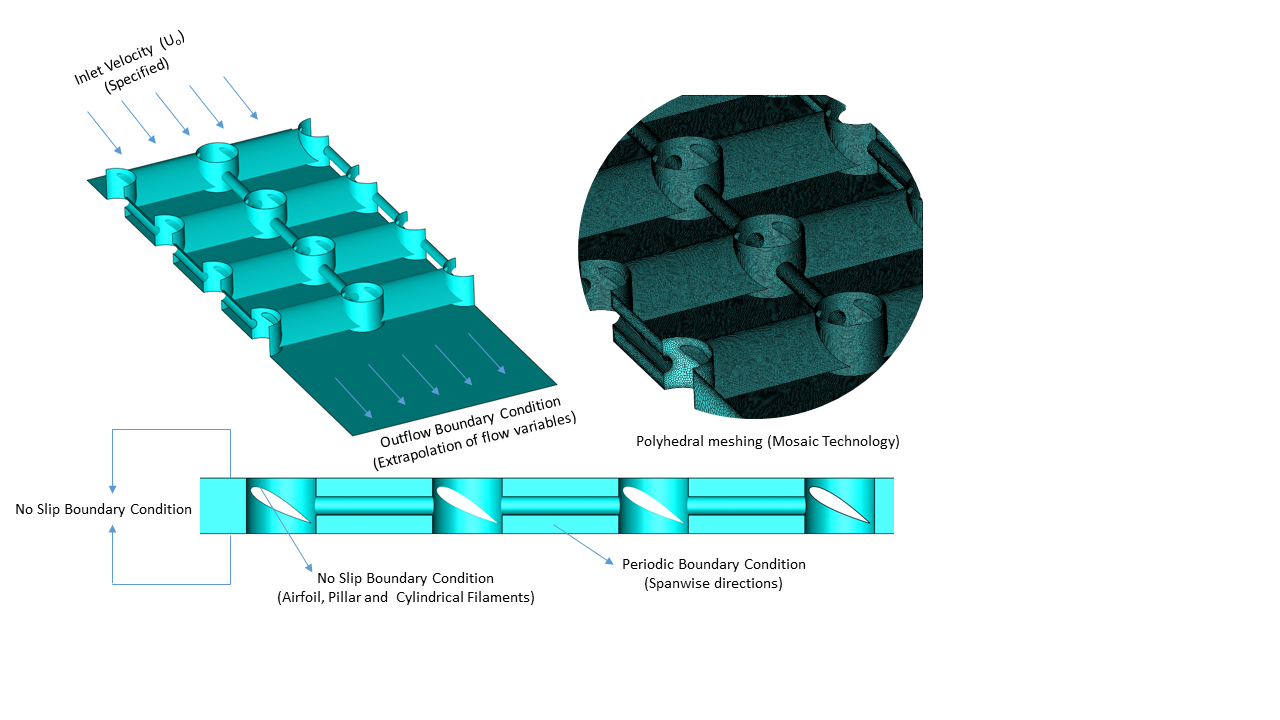


**Fig. S1**. Computational domain extracted from the computer aid design (CAD) along with boundary conditions and mesh used in the simulation.

**References**

1 Moin, P. & Mahesh, K. Direct numerical simulation: a tool in turbulence research. *Annu. Rev. Fluid Mech.* **30**, 539-578 (1998).

2 Qamar, A., Bucs, S., Picioreanu, C., Vrouwenvelder, J. & Ghaffour, N. Hydrodynamic flow transition dynamics in a spacer filled filtration channel using direct numerical simulation. *J. Membr. Sci.* **590**, 117264 (2019).

3 ANSYS. ANSYS Fluent 19.2, Meshing User's Guide, Inc. Southpointe, Canonsburg, PA. (2019).

4 ANSYS. ANSYS Release 19.0, ANSYS Fluent-Theory Guide, Inc. Southpointe, Canonsburg, PA. (2019).
